# Natural Killer Cell Receptor Genes in Camels: Another Mammalian Model

**DOI:** 10.3389/fgene.2019.00620

**Published:** 2019-07-02

**Authors:** Jan Futas, Jan Oppelt, April Jelinek, Jean P. Elbers, Jan Wijacki, Ales Knoll, Pamela A. Burger, Petr Horin

**Affiliations:** ^1^Department of Animal Genetics, Faculty of Veterinary Medicine, University of Veterinary and Pharmaceutical Sciences, Brno, Czechia; ^2^RG Animal Immunogenomics, CEITEC-VFU, University of Veterinary and Pharmaceutical Sciences, Brno, Czechia; ^3^National Centre for Biomolecular research, CEITEC-MU, Faculty of Science, Masaryk University, Brno, Czechia; ^4^Research Institute for Wildlife Ecology, Department of Integrative Biology and Evolution, Vetmeduni Vienna, Vienna, Austria; ^5^Department of Animal Morphology, Physiology and Genetics, Faculty of Agronomy, Mendel University in Brno, Brno, Czechia; ^6^RG Animal Immunogenomics, CEITEC-MENDELU, Mendel University in Brno, Brno, Czechia

**Keywords:** camelid, leukocyte receptor complex, natural killer complex, SNP, microsatellites

## Abstract

Due to production of special homodimeric heavy chain antibodies, somatic hypermutation of their T-cell receptor genes and unusually low diversity of their major histocompatibility complex genes, camels represent an important model for immunogenetic studies. Here, we analyzed genes encoding selected natural killer cell receptors with a special focus on genes encoding receptors for major histocompatibility complex (MHC) class I ligands in the two domestic camel species, *Camelus dromedarius* and *Camelus bactrianus*. Based on the dromedary genome assembly CamDro2, we characterized the genetic contents, organization, and variability of two complex genomic regions, the leukocyte receptor complex and the natural killer complex, along with the natural cytotoxicity receptor genes *NCR1*, *NCR2*, and *NCR3*. The genomic organization of the natural killer complex region of camels differs from cattle, the phylogenetically most closely related species. With its minimal set of *KLR* genes, it resembles this complex in the domestic pig. Similarly, the leukocyte receptor complex of camels is strikingly different from its cattle counterpart. With *KIR* pseudogenes and few *LILR* genes, it seems to be simpler than in the pig. The syntenies and protein sequences of the *NCR1*, *NCR2*, and *NCR3* genes in the dromedary suggest that they could be human orthologues. However, only *NCR1* and *NCR2* have a structure of functional genes, while *NCR3* appears to be a pseudogene. High sequence similarities between the two camel species as well as with the alpaca *Vicugna pacos* were observed. The polymorphism in all genes analyzed seems to be generally low, similar to the rest of the camel genomes. This first report on natural killer cell receptor genes in camelids adds new data to our understanding of specificities of the camel immune system and its functions, extends our genetic knowledge of the innate immune variation in dromedaries and Bactrian camels, and contributes to studies of natural killer cell receptors evolution in mammals.

## Introduction

Camels (*Camelus* spp.) represent an important genus for a number of reasons. Due to their adaptation to desert or semi-desert regions, Old World camels tolerate harsh conditions, which are inhospitable for many livestock species, including extreme temperatures and prolonged periods without access to food and water (reviewed in Jirimutu et al., 2012). As a result, they are of socioeconomic importance across the Middle East, Northern Africa, and much of Asia, where they are used for meat, milk, hides, transportation, and sport. The significance of camels as a sustainable livestock species is likely to continue, as many regions face increased temperatures and desertification as a result of climate change (Megersa et al., 2014; Watson et al., 2016). Concurrently, recent trends towards intensive production and the movement of camel production to peri-urban settings are altering the pathogen pressures to which these animals are exposed (Abdallah and Faye, 2013).

Camels are also of importance with respect to a number of specific infectious diseases. For example, dromedaries (*Camelus dromedarius*) are a natural host of Middle East respiratory syndrome coronavirus, and transmission of the virus from camels to humans has been confirmed (Gossner et al., 2014; Hemida et al., 2017). Interestingly, significant differences exist between dromedaries and Bactrian camels (*Camelus bactrianus*) with regard to their susceptibility to foot and mouth disease (FMD), one of the most costly diseases of production animals worldwide; i.e., dromedaries are not susceptible to FMD and do not transmit infection (Wernery and Kinne, 2012). Furthermore, the immune system of camels has several unusual features. Notable among these is the presence of homodimeric heavy chain antibodies (Hamers-Casterman et al., 1993), not known to occur in any other mammalian family, which have both potential and realized applications in a variety of research, diagnostic, and therapeutic settings (Muyldermans et al., 2009; De Meyer et al., 2014; Steeland et al., 2016). The persistence of uniquely organized ileal Peyer’s patches into adulthood of the dromedary (Zidan and Pabst, 2008) is another example. Additionally, productively rearranged dromedary T-cell receptor delta variable (*TRDV*) (Antonacci et al., 2011) and T-cell receptor gamma variable (*TRGV*) (Vaccarelli et al., 2012) genes undergo somatic hypermutation to generate a diversified repertoire of these genes. This mechanism has not been documented for T-cell receptor genes in other mammalian species and appears to compensate the more limited repertoire of *TRDV* and *TRGV* genes found in camels relative to other Artiodactyls (Ciccarese et al., 2014). A further atypical aspect of the camelid immunogenome is the unusually low genetic diversity of the major histocompatibility complex (MHC) of the three species of Old World camels in both class I (Plasil et al., 2019) and class II genes (Plasil et al., 2016). The immunological characterization of cellular components of the camel immune system is scarce mainly due to the small number of cross-reacting monoclonal antibodies raised against leukocyte antigens of humans (Hussen et al., 2017), bovines, and/or other related species (Mossad et al., 2006) available. This is also one of the reasons why natural killer cells and their functions in camelids have not been studied so far.

Natural killer (NK) cells constitute a heterogeneous lymphocyte population (Allan et al., 2015) involved primarily in innate immune responses against intracellular pathogens and tumor cells. They also influence adaptive immune responses *via* the production of cytokines (Vivier et al., 2011) and crosstalk with dendritic cells (Hamerman et al., 2005), play a role in placentation (Parham and Moffett, 2013), and contribute to the recognition of allogeneic cells. The diversity of the NK cell receptor repertoire is essential to the performance of these multiple functions. The integration of activating and inhibitory signals originating from various surface receptors determines the activation status of an individual NK cell, providing the capacity to discriminate between self and non-self or altered self (Lanier, 1998).

Characterization of genes underlying receptors on the NK cell surface can significantly contribute to our understanding of the functional heterogeneity of NK cells. Among them, NK cell receptors (NKR) of several gene families bind polymorphic MHC class I or MHC class I-like molecules to mediate NK cell function. Due to functional relationships between MHC and NKR molecules, the underlying genes and genomic regions represent an important biological model in terms of their co-evolution in the context of pathogen pressures, disease, and survival (Guethlein et al., 2015; Carrillo-Bustamante et al., 2016). However, the current knowledge of mammalian NKR genes, in comparison with that of MHC regions, is rather fragmentary. Two major genomic complexes encoding NKR, the natural killer complex (NKC) and the leukocyte receptor complex (LRC), have been identified in mammalian genomes. Genes in the NKC represent receptors with C-type lectin-like extracellular domains; genes in the LRC code receptors with extracellular ligand-binding domains belong to the immunoglobulin superfamily (Trowsdale et al., 2001). Despite these structural differences, some receptor families of both complexes are able to fulfil the same functions in terms of MHC class I recognition, downstream signaling, and mediation of NK cell activation/inhibition. To accomplish these ends, different NKR gene families expanded and diversified in different mammalian species, representing an example of convergent evolution in mammals (Kelley et al., 2005; Guethlein et al., 2015). Two immunologically well-defined species, humans and mice, have expanded structurally unrelated receptor families: humans use the killer-cell immunoglobulin-like receptors (KIR) and leukocyte immunoglobulin-like receptors (LILR), both encoded within the LRC, whereas in mice the killer-cell lectin-like receptor (KLR) genes of one family (*Klra*, formerly *Ly49*) are expanded in the NKC. A common sign of these expanded gene families, along with allelic variation of members, is haplotypic variation in the number of genes and pseudogenes in populations/strains of same species (Marsch et al., 2003; Schenkel et al., 2013). The gene content of the LRC (human vs. primates) and NKC (mouse vs. rat) is known to vary even between closely related mammalian species and families; as a result, knowledge of NKR genes in a number of important species remains fragmentary or missing. Significant differences have been shown to exist within Artiodactyls, as for example between cattle and pigs (Sanderson et al., 2014; Schwartz et al., 2017; Schwartz and Hammond, 2018), but knowledge of the genes underlying camel NKR is lacking.

In the context of our work on the camelid immunogenome, the objective of this study was to characterize the genomic content of NKC and LRC with special focus on genes encoding natural killer cell receptors for MHC class I ligands in the two domestic camel species, *C. dromedarius* and *C. bactrianus*.

## Materials and Methods

### Genomic Resources

Based on our new assembly CamDro2 of the *C. dromedarius* genome (Elbers et al., 2019), we characterized the NKC and LRC genomic regions and three natural cytotoxicity receptor genes (*NCR1*, *NCR2*, and *NCR3*). Their gene contents and organization were compared to the National Center for Biotechnology Information (NCBI) reference genomes for *C. dromedarius* (NCBI Genome accession code GCA_000767585.1) assembly PRJNA234474_Ca_dromedarius_V1.0, *C. bactrianus* (GCA_000767855.1) assembly Ca_bactrianus_MBC_1.0, and *Vicugna pacos* (GCA_000164845.3) assembly Vicugna_pacos-2.0.2. The selected orthologous genes were searched in two genomes of domesticated Artiodactyl species, cattle *Bos taurus* (GCA_000003055.5) assembly Bos_taurus_UMD_3.1.1 and pig *Sus scrofa* (GCA_000003025.6) assembly Sscrofa11.1. Various individual genomes for *C. dromedarius* (NCBI BioProject accessions: PRJNA269274, PRJNA269961, and PRJNA310822) and *C. bactrianus* (PRJNA183605 and PRJEB407) were searched in publicly available whole-genome shotgun contigs for candidate microsatellite markers. Likewise, individual whole genome sequencing reads for *V. pacos* (PRJNA233565 and PRJNA340289) were used to estimate single-nucleotide polymorphism (SNP) variability in selected genes of alpacas.

### Annotation of Selected Genes in *C. dromedarius*

Alongside automatic computational annotation of genes in CamDro2 (see Elbers et al., 2019), selected unrecognized genes for NK receptors were manually annotated in the NKC and LRC genomic regions. First, we searched the *C. dromedarius* NCBI reference genome by tblastn algorithm of NCBI’s BLAST^®^^1^ for orthologous protein sequences to killer-cell lectin-like receptors recently identified in cattle as *KLR* genes (Schwartz et al., 2017). Second, the *ab initio* messenger RNA (mRNA) models complementary DNA (cDNA) for corresponding genes of all *KLR* gene lineages in the dromedary reference genome were inspected for completeness using NCBI’s conserved domain database CDD search^2^ and TMHMM Server v.2.0^3^ for prediction of transmembrane helices in predicted proteins. The cDNA for *KLRE* was incorrect; thus, the cattle sequence (Schwartz et al., 2017) was used instead. These cDNA models were aligned against the CamDro2 chromosome 34 sequence using NCBI’s Splign algorithm^4^ and also BLAST^®1^ searched in our full genomic assembly CamDro2. Identified genes were annotated accordingly. The Splign algorithm^4^ was also used on scaffolds for the dromedary, Bactrian camel, and alpaca NCBI reference genomes.

The killer-cell immunoglobulin-like receptor *KIR* genes and leukocyte immunoglobulin-like receptor *LILR* genes were searched on CamDro2 chromosome 9 by the tblastn algorithm^1^. Individual immunoglobulin-like (Ig-like) domains and the cytoplasmic tail of Bactrian 3-domain receptor LILR (XP_010960360) served as query sequences. Orthologous and paralogous sequences were found. The corresponding genomic and predicted cDNA sequences were retrieved from the NCBI reference genomes for both camel species. The full length *KIR* and *LILR* cDNAs were cross-aligned with adequate scaffolds of reference genomes using Splign^4^. The predicted protein sequences were screened with CDD^2^ and TMHMM Server v. 2.0 search^3^. The gene sequences were BLAST^®1^ searched in CamDro2. Identified full-length genes were annotated, and gene fragments were recorded.

The natural cytotoxicity receptor genes *NCR1*, *NCR2*, and *NCR3* were BLAST^®^ searched in the full assembly CamDro2 based on annotation of the dromedary NCBI reference genome. Although there exist numbers of alternatively spliced variants for each gene/protein, we focused on cDNA models of the longest variant (**Table 1**). The predicted dromedary, Bactrian camel, and cattle cDNA for *NCR3* were incomplete; therefore, we used the sequence predicted from the white-tailed deer *Odocoileus virginianus texanus* instead.

**Table 1:** Number of predicted protein sequences differing by at least one amino acid residue.

Locus	mRNA model	Alias for protein	*Camelus dromedarius*	*Camelus bactrianus*	*Vicugna pacos*
*KLRA*	XM_010986205	Ly49	2	2	5
*KLRC1*	XM_010986199	NKG2A	3	3	5
*KLRC2*	XM_010986200	NKG2C	2	1^§^	5
*KLRD*	XM_010986196	CD94	3	1	3
*KLRE*	KX611578.1		2	1	5^§^
*KLRI*	XM_010986247		1	1	3
*KLRJ*	XM_010986202		2*	1*	5*
*KLRK*	XM_010986198	NKG2D	2	2	4
*KIRDP*	XM_011000160		NA	NA	NA
*KIR3DL*	XM_014563526	KIR3DL1, CD158	7*	3*	7
*LILRB1*	XM_010999297		3	1	7
*LILRB2*	XM_010961869^+^		6	2*	ND
*LILRB3*	XM_010961881^+^		4	5	ND
*LILRA 2-Ig*	XM_010961868		3	1	ND
*LILRA 4-Ig*	XM_011000157		3	4	ND
*NCR1*	XM_010961883	NKp46, CD335	2	3^§^	3
*NCR2*	XM_010973934	NKp44, CD336	2^§^	3	3
*NCR3*	XM_020897188	NKp30, CD337	2*	2*	3*

*Alleles predicted for a pseudogene; ^§^Includes allele with premature stop codon; ^+^Modified to represent all exons; NA, not applicable; ND, not determined.

During the process of manual annotation, we also characterized the NKC and LRC regions, and comparisons were made between *C. dromedarius*, *C. bactrianus*, and *V. pacos*.

To allow comparisons with other studies and identification of orthologues, a standardized systematic nomenclature of NKR genes (Schwartz et al., 2017) was used. However, when referring to original reports on human or mouse genes, both the original and standard gene names and symbols were used.

### Amplification and Next-Generation Sequencing

The gene-specific primers encompassing full-length genes with flanking sequences were designed on NCBI *C. bactrianus* gene sequences using Primer-BLAST^5^ and checked for specificity against reference genomes of both camel species. The list of primer pairs used is available in **Supplementary Table 1**. Various compositions of PCRs and adequate thermal profiles used are summarized in **Supplementary Table 2**. PCR products were checked by 0.5% agarose gel electrophoresis and quantified by Invitrogen^™^ Qubit^™^ Fluorometer using Qubit^™^ dsDNA BR Assay Kit (Thermo Fisher Scientific, Waltham, MA, USA). They were kept frozen at −20°C until massive parallel sequencing. Each individual’s long-range amplicon of genes under study was indexed separately during library preparation using the NexteraXT DNA Library Preparation Kit (Illumina, San Diego, CA, USA) and sequenced on a MiSeq^™^ System (Illumina, San Diego, CA, USA) platform using the MiSeq^™^Reagent Kit v2 (500 cycles) according to manufacturer’s protocol in different runs. The quality of the raw sequencing reads was checked using FastQC^6^. Low quality read ends were removed by Trimmomatic (Bolger et al., 2014) (SLIDINGWINDOW:4:15). Only reads longer than 150 bp were used for the mapping by BWA-MEM (Li, 2013). The alignment was post-processed using Samtools (Li et al., 2009) (sorting and conversions), GATK (DePristo et al., 2011) (indel realignment), and Picard^7^ (PCR duplicates removal). Further, only mappings with the minimal mapped length of 70 bp, a maximum of 5% soft-clipping, and a maximum of 10% mismatches were kept using NGSUtils (Breese and Liu, 2013) and BBMap^8^.

### Microsatellite Markers

The repetitive sequences of di-, tri-, and tetra-nucleotides were searched in the NKC and LRC of the Bactrian camel NCBI reference genome by RepeatMasker^9^. Candidate microsatellites (msats) were identified by BLAST^®^ search of repetitions flanked with 100 bp sequences in whole-genome shotgun contigs from three dromedaries and two Bactrian camels. The most diverse sequences with unique occurrence in genome were chosen, and primers were designed in OLIGO Primer Analysis Software v.4.0 (Molecular Biology Insights, Colorado Springs, CO, USA). Primer specificity was verified against the NCBI reference genomes of both camel species using NCBI’s Primer-BLAST^5^. The PCR conditions were optimized for six msats, finalizing in one 5-plex (*CZM025*-*CZM029*) and one single (*CZM030*) PCR protocol. Reaction compositions were as follows: 1.0 μl 10 × Taq Buffer (Top-Bio, Prague, Czech Republic), 0.5 U CombiTaq DNA polymerase (Top-Bio, Prague, Czech Republic), 200 µM each dNTP (Thermo Fisher Scientific, Waltham, MA, USA), 0.1 μl of each primer of concentration 10 μM (**Table 2**), and 50 ng of genomic DNA. PCR reaction mix was supplemented with PCR grade H_2_O (Top-Bio, Prague, Czech Republic) to a final volume of 10.0 μl. The thermal cycler ABI Verity 96 Well (Applied Biosystems, Foster City, CA, USA) was used for amplification. The thermocycling conditions consisted of initial denaturation at 95°C for 3 min; 30 cycles of denaturation at 95°C for 30 s, annealing at 56°C (64°C for *CZM030*) for 30 s, and elongation at 72°C for 30 s; and final elongation at 72°C for 60 min and holding at 7°C. All markers were then tested by fluorescent fragment analysis using Applied Biosystems^®^ ABI PRISM 3500 and sized with GeneScan^™^ 500 LIZ^®^ Size Standard (Thermo Fisher Scientific, Waltham, MA, USA). The data obtained from the fragment analyzer were evaluated using the GeneMapper^®^ software v.4.1 (Thermo Fisher Scientific, Waltham, MA, USA).

**Table 2:** Characteristics of microsatellite markers in natural killer complex (NKC) and leukocyte receptor complex (LRC) regions.

Marker	Type of repetition	*C. dromedarius* amplicon size	Forward primer Reverse primer
*CZM025*	(AC)n	184–210 bp	6-FAM-CCCACAGGCTGTTCCTCAAA-3′ 5′-TGTCCTGGTTATGGGAAGATGG-3′
*CZM026*	(GT)n	216–236 bp	NED- CTTCCAAATGCACCTGAAACATC-3′ 5′-TGACTTCAAGGGAATGCCTCAA-3′
*CZM027*	(TG)n	233–247 bp	6-FAM-GGCTGGACAGTGCAAATTTTACC-3′ 5′-GCACCATCTCTGGAGGCTAAGAG-3′
*CZM028*	(AC)n	95–113 bp	NED- TAATACTCGCCATCCTTCTGCCT-3′ 5′-GAGACCCTCCGGTGTAGAAAGC-3′
*CZM029*	(AC)n	169–193 bp	NED- ATCATGTCAGCATTGCTTTGGAA-3′ 5′-CATGTGTCCTGACGCTGGAA-3′
*CZM030*	(CA)n	167–187 bp	6-FAM-CCGTCAGCTGGAAATTTGTCTCT-3′ 5′-AGAGTCAGGAGGGCTTCTAGGCTA-3′

### Estimation of Genetic Variability in Camels and Alpacas

The dromedary DNA samples in this study were transferred from previous projects [Austrian Science Fund (FWF)P1084-B17 and P24706-B25; PI: P. Burger] and originated either from plucked hair, ethylenediaminetetraacetic acid (EDTA) blood collected commensally on Whatman FTA^®^ cards (Sigma-Aldrich, Vienna, Austria) during routine veterinary controls, or from DNA extracts sent by collaborators under bilateral agreements. Samples were imported with permits from the Austrian Ministry of Labour, Social Affairs, Health and Consumer Protection. All the Bactrian camel samples were collected commensally during veterinary procedures for previous research projects (GACR 523/09/1972; PI: P. Horin). Details about the samples are provided in the **Supplementary Table 3**.

Selected genes of the NKC and LRC regions were genotyped by targeted resequencing of long-range PCR amplicons in both camel species. For comparison, individual genotypes in the same batch of genes except *LILR* genes were acquired for alpacas by data mining.

Two panels of 10 animals were created from collections of samples originating from various populations. The *C. dromedarius* panel encompassed individuals coming from Jordan (Irbid), Iran, Saudi Arabia (Magaheem and Wadda), Canary Islands, UAE (Dubai), Kenya, Sudan, Nigeria, and Kazakhstan. The genomic DNA was previously isolated by phenol-chloroform extraction and kept frozen at −20°C. The C. *bactrianus* panel consisted of individuals from three Mongolian regions (Bayan Ovoo, Galshar, and Norovlin). The genomic DNA was isolated from frozen archived whole blood samples by NucleoSpinBlood^©^ Kit (Macherey-Nagel, Düren, Germany) according to the manufacturer’s protocol. The genes of interest were isolated by PCRs on genomic DNA, obtained amplicons were indexed to track individual samples, and then were sequenced in multiple Illumina next-generation sequencing (NGS) runs and mapped to adequate reference sequence for amplicon (see above).

A panel of four alpacas was created from publicly available whole-genome sequencing projects. The raw data of Illumina NGS runs were downloaded from the European Nucleotide Archive database^10^ (ENA accession numbers SRR1552593-1552609, SRR4095109, SRR4095110, and SRR4095135). The quality was checked using FastQC^6^ and Kraken package (Davis et al., 2013). Adapter and quality (Phred < 15) trimming was performed by Cutadapt (Martin, 2011). BWA-MEM (Li, 2013) was used for the alignment, and the alignments were post-processed by Samtools (Li et al., 2009) (sorting and conversions), GATK (DePristo et al., 2011) (indel realignment), and Picard^7^ (PCR duplicates removal). Alignments were further filtered using NGSUtils (Breese and Liu, 2013) and BBMap^8^ for maximum soft-clipping (5%), maximum number of mismatches (10), minimum mapped length (35 bp), maximum soft-clipping (5%), and minimum mapping quality (MAPQ 40). The reference sequences for mapping were retrieved from the *V. pacos* NCBI reference genome. Most *V. pacos* sequences were framed by the primer sequences used in camels.

### Data Analysis

The alignments of reads to the reference sequence were inspected using IGV software^11^. The variable positions (variant in homozygous state) and confirmed sequence variants (variant detected in heterozygous state) were treated as SNPs. They were written to consensus sequences for each animal using IUPAC nucleotide ambiguity codes in BioEdit, version 7.2.6. (Hall, 1999) along with insertions/deletions, and sequences from same animal species were manually aligned. The number of SNPs was counted using DnaSP version 5.10 program (Librado and Rozas, 2009), and frequency was calculated as percentage. The cDNA sequences were *in silico* extracted in BioEdit v.7.2.6, based on mRNA models for each gene (**Table 1**) and checked by Splign^4^ for completeness. Haplotypes of each diploid individual were reconstructed for every panel and gene (cDNA) separately using PHASE (Stephens and Donnelly, 2003) algorithm in DnaSP v.5.10. The coding sequences were extracted in BioEdit v.7.2.6. The number of SNPs was counted in DnaSP v.5.10, and the percentage of coding sequence length calculated.

Amino acid sequences were deduced from coding sequences in BioEdit v.7.2.6. The manually aligned predicted protein sequences were compared. Sequences differing in at least one amino acid position were numbered and designated as different alleles of a particular gene.

A phylogenetic analysis of sequences obtained by long-range PCR or data mining was done separately for NKC (C-type lectin-like) and LRC (immunoglobulin-like) genes. The nucleotide coding region sequences were aligned by ClustalW Multiple alignment algorithm in BioEdit v.7.2.6. One haplotype per gene was chosen for each species to represent the respective loci of the dromedary, Bactrian camel, and alpaca. Corresponding cattle and pig sequences retrieved from NCBI’s GenBank^12^ were used for a comparison. The maximum likelihood phylogenetic trees were constructed in MEGA5 (Tamura et al., 2011) based on the Tamura 3-parameter model and the partial deletion method (95% cutoff) with 100 bootstrap repetitions (Tamura, 1992).

## Results

**Figure 1:**
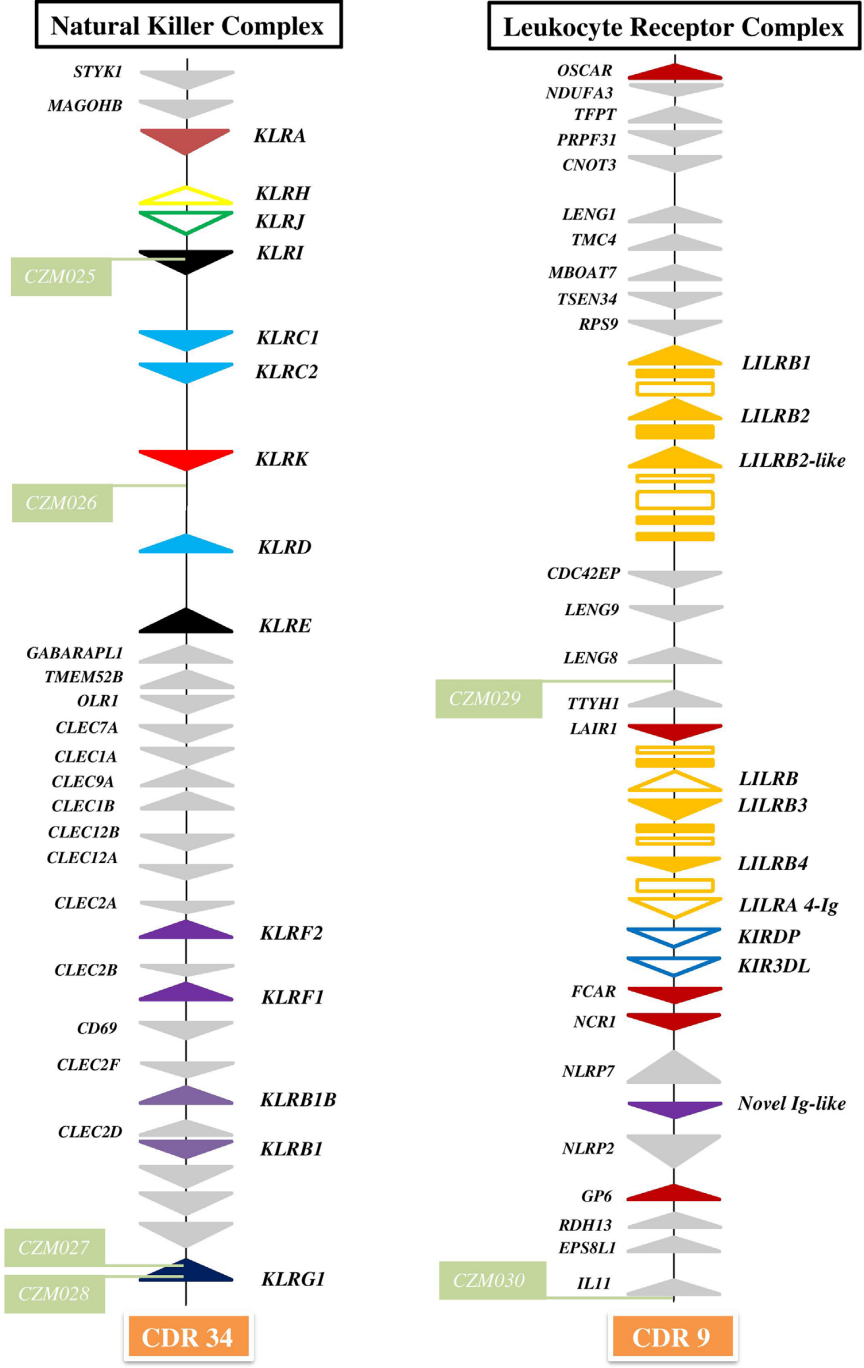
Organization of genomic regions encoding NK cell receptors in dromedary camel. The NKC was delineated by *STYK1* and *KLRG1* genes on chromosome 34 (CDR34) of CamDro2 between 11.61 and 12.50 Mb. *KLR* genes are represented as *solid color triangles*, *KLR* pseudogenes as *empty color triangles*, and lectin-like *CLEC* or non-lectin genes as *solid grey triangles*. The LRC was found between the *OSCAR* and *IL11* genes on chromosome 9 (CDR9) in the region 63.00–72.01 Mb. *LILR* genes are represented as *solid orange triangles*, *LILR* pseudogenes as *empty orange triangles*, immunoglobulin-like domains or cytoplasmic domain gene fragments as *orange rectangles*, *KIR* pseudogenes as e*mpty blue triangles*, other types of Ig-like genes as *solid color triangles*, and different types of flanking genes as *solid grey triangles*. *Green rectangles* mark positions of newly developed microsatellite markers *CZM025*–*CZM30*.

**Figure 2:**
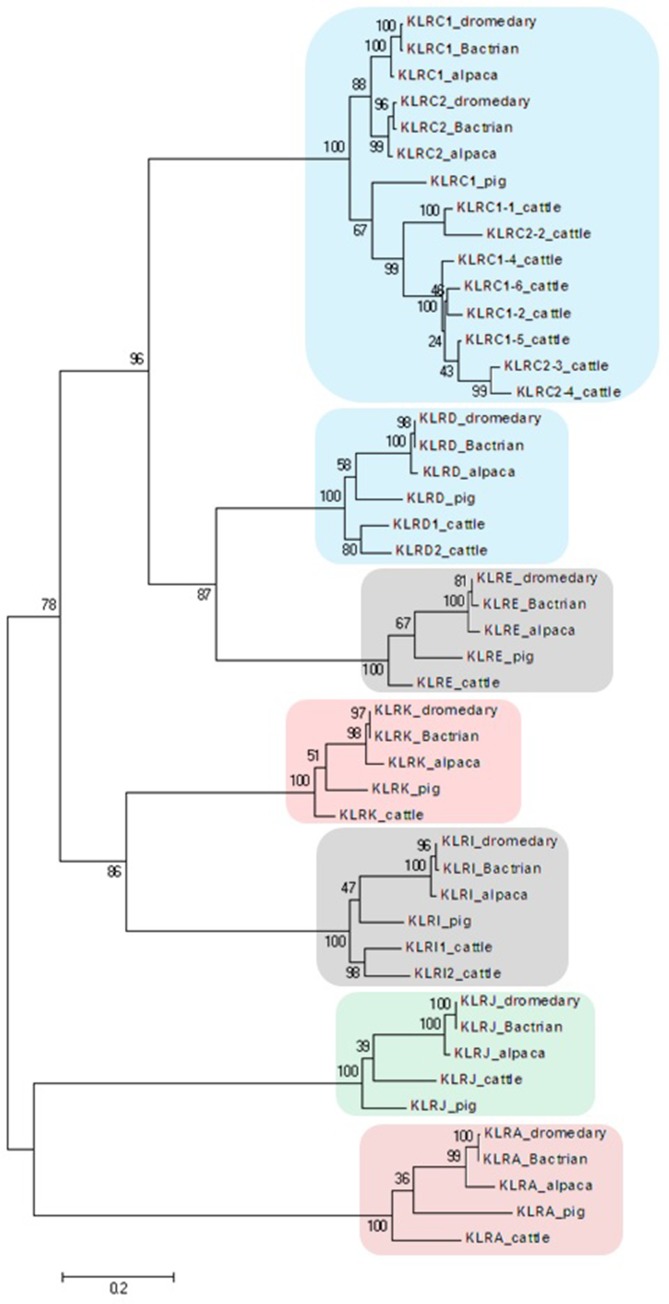
Phylogeny of nucleotide coding sequences for NKC C-type lectin-like genes analyzed by long-range PCR/data mining. The percentage of trees (out of 100 bootstrap replicates), in which the associated sequences clustered together is given at branch nodes. Branch lengths are expressed as the number of substitutions per site. Clusters of genes are highlighted according to the color scheme used in Figure 1. Haplotypes generated in this study were chosen (one per gene) to represent loci of *Camelus dromedarius* (dromedary), *Camelus bactrianus* (Bactrian), and *Vicugna pacos* (alpaca). Comparisons were made to *Bos taurus* (cattle) sequences retrieved from GenBank (accession numbers: KX611576, KX611577, KX611578, KX698607, NM_174376.2, NM_001075139.1, NM_001098163.1, and NM_001168587.1) and *Sus scrofa* (pig) sequences (accession numbers: NM_213813.2, NM_214338.1, XM_005655677.3, XM_005655679.3, XM_013988381.2, XM_013988416.2, and XM_021092357.1).

**Figure 3:**
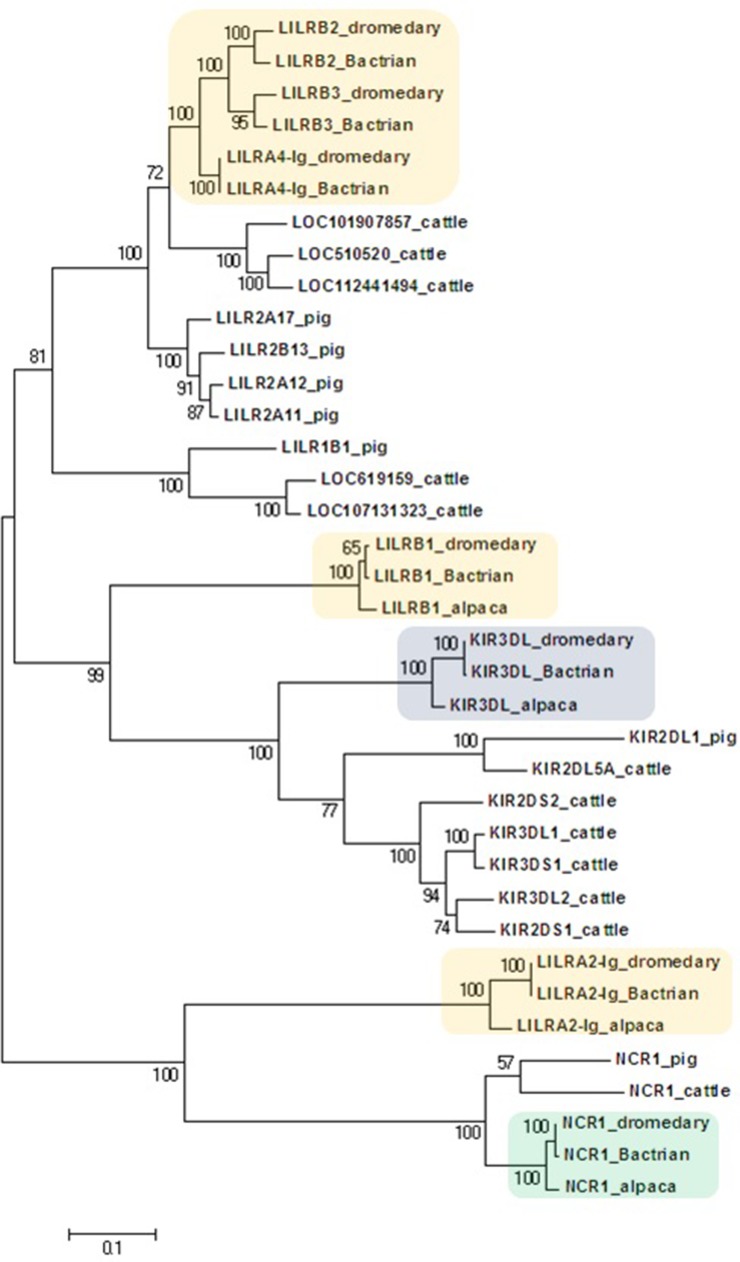
Phylogeny of nucleotide coding sequences for LRC immunoglobulin-like genes analyzed by long-range PCR/data mining. The percentage of trees (out of 100 bootstrap replicates) in which the associated sequences clustered together is given at branch nodes. Branch lengths are expressed as the number of substitutions per site. Haplotypes generated in the study were chosen (one per gene) to represent loci (*colored*) of *Camelus dromedarius* (dromedary), *Camelus bactrianus* (Bactrian), and *Vicugna pacos* (alpaca). The nomenclature for the camelid *LILR* gene family is provisional and will change when a complete assembly of this region is available. Some alpaca *LILR* genes were not included due to an incomplete resolution of this family in the reference genome. The alpaca’s *LILRA 2-Ig* sequence was retrieved from GenBank (accession number XM_015252448.1). A selection of *Bos taurus* (cattle) sequences (accession numbers: NM_174740.2, NM_181451.1, NM_183365.1, NM_001008415.1, NM_001097567.1, NM_001098089.1, XM_005201067.4, XM_024978801.1, XM_024978806.1, XM_024978818.1, XM_024978824.1, and XM_024978827.1) and all functional *Sus scrofa* (pig) sequences (accession numbers: NM_001113218.1, NM_001123143.1, NM_001128451.1, XM_003134173.4, XM_013998762.2, XM_021094960.1, and XM_021094977.1) were used for comparison.

The general organization of the two genomic regions, the natural killer complex (NKC) and the leukocyte receptor complex (LRC), containing genes and gene families encoding the NK cell receptors annotated based on the dromedary genome assembly CamDro2, was established and is represented in **Figure 1**. The phylogenetic trees of the genes analyzed are shown in **Figures 2** and **3** for NKC and LRC genes, respectively. A summary of the allelic variants of the predicted proteins for the genes genotyped in dromedaries and Bactrian camels is given in **Table 1**. The alignments of amino acid sequences with depictions of their protein domains are provided in the **Supplementary Figures S1–S3**. An overview of SNPs of the full genes and coding sequences is found in **Supplementary Table 4**. Protein homology of selected receptors in dromedary relative to Bactrian camel, alpaca, cattle, and pig orthologues is summarized in **Supplementary Table 5**.

### Natural Killer Complex

The NKC region encompassing approximately 0.9 Mbp was localized on chromosome 34 of CamDro2. Twenty-six genes encoding receptors with the C-type lectin-like domain (CTLD) of different lineages were identified in this region. No expansion of any *KLR* gene family was observed in the dromedary genome. Most *KLR* genes clustered at one end of the region. This cluster contains five functional genes (*KLRA*, *KLRD*, *KLRE*, *KLRI*, and *KLRK*), two functional members of the *KLRC* family, and two pseudogenes (*KLRH* and *KLRJ*). *KLRC* is the only gene family with two members sharing the same CTLD but signaling oppositely: *KLRC1* codes for an inhibitory receptor, while *KLRC2* encodes an activating receptor. Members of three families (*KLRB*, *KLRF*, and *KLRG*) are located at the opposite end of the NKC region, separated from each other by a group of C-type lectin (*CLEC*) genes. Two members of each of these families were found in the CamDro2 dromedary genome. The genes *KLRB1* and *KLRB1B* have the standard structure of inhibitory receptors with a cytoplasmic tail containing an immunoreceptor tyrosine-based inhibitory motif (ITIM). Genes encoding their putative ligands (*CLEC2D* and *CLEC2F*, respectively) were found in the vicinity. Similarly, the genes *KLRF1* (NKp80) and *KLRF2* (NKp65) encoding activating receptors are located in close proximity of genes coding for their predicted ligands, AICL and KACL (*CLEC2B* and *CLEC2A*, respectively). *KLRG1* encoding an inhibitory receptor marks the boundary of the NKC. *KLRG2* was found outside of NKC, on chromosome 7.

While in the *C. dromedarius* NCBI reference genome the NKC is split into two scaffolds (NW_011591409, NW_011591669), it is contained within a single scaffold (NW_011511552) in the *C. bactrianus* NCBI reference genome. The gene content and gene orientations are the same in both genomes. The only exception is the presence of a premature stop codon in the Bactrian *KLRC2* sequence, which thus seems to be a pseudogene.

### Variability of NKC Receptors

Since no expansion of *KLR* gene families was observed, we focused on the allelic variation of inhibitory receptors supposed to recognize MHC class I ligands. Due to their poor quality, some samples were not successfully amplified by long-range PCRs. Because of an apparent mixed ancestry (*C. dromedarius* X *C. bactrianus*) of one *C. dromedarius*, heterozygous sequences of mixed origin were removed. Consequently, different numbers of genotypes were retrieved for different genes as indicated in **Supplementary Table 4**. Despite polymorphisms existing in the genomic and predicted mRNA sequences (**Supplementary Table 4**), none of the tested genes were found to have more than three protein variants. Five of the eight tested genes in *C. bactrianus* were monomorphic on the protein level.

*KLRA* encodes an inhibitory receptor with one ITIM signaling motif in its cytoplasmic tail and a relatively long extracellular stalk region (over 70 amino acids). Two variants of this receptor molecule were predicted in *C. dromedarius*, sharing the same CTLD but differing by one amino acid residue in the cytoplasmic tail. A KLRA variant with the same CTLD occurs frequently in *C. bactrianus*. A second variant of Bactrian KLRA differs by nine amino acids (one in the cytoplasmic tail, four in the stalk, and four in the CTLD).

*KLRC1* codes for an inhibitory receptor with two ITIMs. Three variants of the KLRC1 protein were identified in each camel species. One KLRC1 variant was shared by both camel species, and two additional variants in each species differed by only one amino acid. Only two different CTLD variants were present in each species.

*KLRC2* codes for an activating receptor with a charged amino acid residue (lysine) in the transmembrane domain. In *C. dromedarius*, *KLRC2* appears to encode two variants of a functional receptor. In *C. bactrianus*, this gene seems to be monomorphic with a premature stop codon shortly after the origin of translation.

The *KLRD* gene product consists of a CTLD with a short stalk and a transmembrane domain with no signaling motif. For KLRD, one protein sequence common to both camel species was observed, with two additional variants found in *C. dromedarius*, differing by one amino acid each. All three camel genes, *KLRC1*, *KLRC2*, and *KLRD*, have codons for cysteine residues in the stalk region of the protein, allowing formation of disulfide links and of heterodimers KLRD/KLRC1 and KLRD/KLRC2.

Another pair of presumably interacting receptors forming noncovalent heterodimers is KLRE/KLRI. The striking features of Old World camelid *KLRE* are the presence of sequence for an ITIM in the cytoplasmic tail of the protein and the existence of a second variant in *C. dromedarius* with a duplication of six amino acid residues in the CTLD. In *C. bactrianus*, *KLRE* encodes only one variant of the protein sequence.

The *KLRI* gene showed very limited polymorphism in both camel species at the genomic level, encoding only one protein variant with only one functional ITIM (and one mutated motif) in the cytoplasmic tail of the molecule.

The predicted protein product of the *KLRJ* gene was identical in both camel species. Another sequence variant in the dromedary camel differed by only one amino acid in the stalk region. According to the adopted mRNA model, all these sequences contain a premature stop codon in the CTLD of the protein.

*KLRK* in both camel species codes for a functional activating receptor with a charged amino acid (arginine) in the transmembrane region. Two proteins with variant CTLD were recognized in *C. dromedarius*, while two proteins in *C. bactrianus* share the same CTLD as one of the dromedary KLRK.

In the phylogenetic trees obtained, all NKC genes sequences of both camel species clustered with their putative orthologues in alpaca, cattle, and pig (**Figure 2**).

### Leukocyte Receptor Complex

The LRC region of approximate length 0.7 Mbp was localized to chromosome 9 of CamDro2. Fifteen full-length genes encoding receptors containing immunoglobulin-like (Ig-like) domains of various lineages were identified in the LRC region. Besides two *KIR* pseudogenes containing ITIM domains and an expanded *LILR* gene family, a singular Ig-like receptor was found in the vicinity of *FCAR* and *NCR1*, located between *NLRP7* and *NLRP2*. It comprises two Ig-like domains different from those of the *LILR* and the *KIR* genes and has a long cytoplasmic tail with two ITIMs. It is a novel type of LRC gene, similar to a gene recently identified in pigs (Schwartz and Hammond, 2018). Based on its structure, this inhibitory type of receptor gene may be functional, similarly to pigs.

The expanded family of *LILR* genes is organized in two distinct clusters. The first region spanning approximately 141 kb is located between the genes *RPS9* and *CDC42EP5*. This region contains three putatively functional genes, *LILRB1*, *LILRB2*, and a *LILRB2-like* sequence. *LILRB1* codes for an inhibitory receptor with three Ig-like domains. *LILRB2* and *LILRB2-like* each encode four Ig-like domains and a cytoplasmic domain with ITIMs. Several fragmented sequences containing Ig-like domains were identified within this region as well. The second *LILR* region spanning approximately 127 kb is located between *LAIR1* and the two *KIR* pseudogenes. Three full-length *LILR* genes and a pseudogene were identified in this region. *LILRB3* codes for an inhibitory receptor; it comprises four Ig-like domains, a transmembrane region, and a cytoplasmic tail with two intact ITIMs. *LILRB4* also codes for an inhibitory receptor, but with only two Ig-like domains. In addition, there are a potentially functional activating *LILRA* gene and a *LILRB* pseudogene (containing an ITIM sequence) located in the same region. The predicted LILRA contains four Ig-like domains in its extracellular region but has no signal peptide sequence. The cytoplasmic domain is short and contains no ITIMs. Several fragments with complete or partial Ig-like domains were also found in this region.

In the current *C. dromedarius* NCBI reference genome, the LRC is split amongst at least four scaffolds (NW_011593473, NW_011591120, NW_011595380, and NW_011591711). They matched our CamDro2 assembly in terms of the number and orientation of orthologous non-Ig-like genes recognized by automatic annotation. Single Ig-like genes and two *KIR* pseudogenes, but not expanded *LILR* genes, were unraveled. The Bactrian LRC of NCBI reference genome is contained within a single scaffold (NW_011515311), but assembly of the expanded *LILR* genes is not resolved and *LILRB2-like* is missing. The overall LRC organization in the *C. bactrianus* reference genome is the same as that of the LRC of dromedaries (**Figure 1**).

### Variability of LRC Receptors

Based on PCR and resequencing of representative panels of *C. dromedarius* and *C. bactrianus*, individual genotypes could be successfully identified for most of the genes analyzed. However, the amplification of *KIR3DL* sequences in Bactrian camels provided only limited numbers of sequences (**Supplementary Table 4**). Similar to NKC genes, some sequences from one *C. dromedarius* individual were removed due to their mixed origin.

The *KIR3DL* gene contains a 2-bp deletion in the exon for the third Ig-like domain, causing a frameshift and a premature stop codon. This deletion is identical in both camel species. The locus *KIRDP* contained sequences with premature stop codons and frameshift mutations in both camel species. The same was found in NCBI reference genomes.

Therefore, we assigned *KIRDP* and *KIR3DL* sequences provisionally as pseudogenes in both species. In contrast to the low polymorphism of the NKC receptors, higher numbers of variable amino acid sites were found within KIR3DL. An *in silico* 2-bp insertion resulted in three and seven predicted full-length protein variants in Bactrian camels and dromedaries, respectively (**Table 1**).

*LILRB1* encodes a protein of similar structure to *KIR3DL*, with three constant-type Ig-like domains and two functional ITIMs in its cytoplasmic tail. Unlike other members of the *LILR* family coding for receptors with four Ig-like domains, *LILRB1* has no variable-type Ig-like domain between its first and second domains. Nevertheless, in *C. dromedarius*, only three variants with minor changes in their amino acid sequences were found, and in *C. bactrianus*, this gene appears to be monomorphic.

The gene *LILRB2* of *C. dromedarius* encodes a functional inhibitory receptor with four Ig-like domains and two ITIMs. Six variants recognized in the panel of dromedaries share the same Ig-like domains and differ only by two amino acids in the transmembrane region and one in the cytoplasmic tail. All *LILRB2-like* sequences obtained by PCR were identical to *LILRB2* sequences retrieved from the same dromedaries’ DNAs. In *C. bactrianus*, *LILRB2* encodes two mRNAs with a premature stop codon in the first Ig-like domain and differs by 37–38 amino acid positions from its dromedary counterparts. No PCR products were obtained for *LILRB2-like* from the Bactrian camel panel.

*LILRB3* encodes a receptor with 82% identity (86% similarity) to LILRB2 in *C. dromedarius*. All four protein variants had only two different Ig-like domains (the second and third) with one amino acid change each. Five variants of the Bactrian LILRB3 had 16 inter-species specific positions with another 11 amino acids differing within species.

Sequences of two activating *LILRA* genes were retrieved by PCR. One of them, containing two Ig-like domains, arginine in the transmembrane region, and a long cytoplasmic tail, but with the first ITIM deleted and the second mutated, was provisionally named as *LILRA 2-Ig*. Both camel species shared one variant of the LILRA 2-Ig protein, and two additional variants were present in the dromedary. The second activating gene was designated *LILRA 4-Ig* as it contained four Ig-like domains, arginine in its transmembrane region, and a short cytoplasmic tail with no signaling motif. Three variants of the dromedary and four variants of the Bactrian camel LILRA 4-Ig protein are very similar, differing in only six amino acid positions. One specific variant with an in-frame deletion of 25 amino acids in the third Ig-like domain was shared between species.

The phylogenetic tree constructed for the coding regions of the camel LRC genes (**Figure 3**) and their homologs in alpaca, cattle, and pig revealed three main clusters of genes characterized by the overall structure of the encoded receptor. The first cluster grouped genes with four Ig-like domains receptors (LILRs). The second group was a cluster of genes coding for receptors with three or two Ig-like domains (KIRs and LILRB1). The third cluster was formed by genes encoding receptors with two Ig-like domains (NCR1 and LILRA2-Ig). Within the first cluster, three distinct camel genes, *LILRB2*, *LILRB3*, and *LILRA4-Ig*, clustered with various cattle and pig *LILR* genes. Likewise, various cattle and pig *KIR* genes formed a cluster with camelid *KIR3DL* genes. This cluster was related with the cluster of camelid *LILRB1* genes, while the cluster containing *NCR1* gene sequences was related to the camelid *LILRA2-Ig* genes. As no cattle and/or pig homologs of camelid *LILRB1* and *LILRA2-Ig* genes could be identified in the reference genomes of these two species, they did not appear in the trees constructed.

### The Natural Cytotoxicity Receptors

The predicted proteins of the *NCR1*, *NCR2*, and *NCR3* genes were studied as potentially activating immunoglobulin-like receptors for various ligands different from MHC class I.

The *NCR1* gene is located within the LRC, and the structure of camelid NCR1 is similar to the structure of this gene’s products in other species, with two extracellular Ig-like domains and a charged residue (arginine) in the transmembrane domain, which allows its interaction with activating adaptor proteins. Two allelic variants were identified in each camel species that differed from each other by only one or two amino acids. *C. bactrianus* possessed one additional variant containing a premature stop codon in the first Ig-like domain.

The *NCR2* gene is located on chromosome 20 of CamDro2. It encodes a functional receptor with one extracellular Ig-like domain and a charged residue (lysine) in the transmembrane domain. One allelic variant of the receptor is shared by both camel species. Another variant, found only in *C. dromedarius*, has a premature stop codon in the stem region of the putative molecule. Two additional variants were identified in *C. bactrianus*, differing by one amino acid each.

The *NCR3* gene is also located on chromosome 20, within the MHC region. All sequences in both camel species contained the same two premature stop codons; this gene thus seems to be nonfunctional in camels.

### “*In Silico*” Comparison With *V. pacos*

The alpaca is evolutionarily the most closely related species to the Old World camels. The *V. pacos* NCBI reference genome contains two scaffolds (NW_005882720 and NW_005883060) with *CLEC* and *KLR* genes. The gene content and organization of the alpaca NKC region was found to be very similar to that of the camel NKC, with similarities of amino acid sequences ranging from 88% to 98%. Based on publicly available alpaca genomes, the extent of polymorphism of genomic as well as of protein sequences was higher than in either of the camel species. Five protein variants were predicted for *KLRA*, *KLRC1*, *KLRC2*, and *KLRE*. The amino acid changes were concentrated in the CTLD and the stem of KLRA and in the CTLD in KLRC2. In contrast, they were evenly distributed throughout KLRC1. *KLRE* coded for four functional protein variants with only three different CTLDs, and amino acid changes were concentrated mostly in the cytoplasmic tail. One allele had a 1-bp insertion leading to frameshift and a premature stop codon in the CTLD part of the receptor. The same ITIM motif as in camels was recognized in all five variants. The three variants of KLRD identified differed by one amino acid each in the stem region of the receptor and thus shared the same CTLD. *KLRI* also coded for three variants with the same CTLD, differing only in the cytoplasmic tail. Due to a non-synonymous substitution, a second ITIM was recreated in one of the variants. The five protein variants predicted for *KLRJ* differed in their CTLDs, although all of them contained the same premature stop codon according to the mRNA model. *KLRK* haplotypes coded for a potentially functional activating receptor (arginine in the transmembrane region). Only one amino acid difference was observed in two CTLD types shared by the four KLRK protein variants.

The alpaca LRC region is fragmented amongst numerous scaffolds of the NCBI reference genome. One of them (NW_005882947) contains a four-domain *LILRB* gene, *KIRDP*, *KIR3DL*, *FCAR*, *NCR1*, *GP6*, and a novel Ig-like gene, while another scaffold (NW_005883177) contains *LILRB1*, comprising three Ig domains, and *LILRB*, with four Ig domains. Only fragments of *LILR* genes could be found in the rest of the relevant scaffolds. Like in camels, *KIRDP* contains various frameshifts. In contrast, *KIR3DL* has retained an intact genomic sequence and is thus likely to encode a functional inhibitory receptor, but with only one functional ITIM. The second ITIM is mutated in all protein variants. Seven variants of KIR3DL were identified, differing in 11 amino acid positions in total. These sites are equally distributed throughout the molecule. *LILRB1* also codes for a potentially functional inhibitory receptor with three Ig-like domains and seven identified variants. They differed in 17 positions located in two of the Ig-like domains, the stem and the cytoplasmic tail. The protein homology in comparison with dromedary camel *KIR3DL* and *LILRB1*counterparts reached 93%.

The gene *NCR1* of the alpaca encodes a functional activating receptor with a charged amino acid residue (arginine) in the transmembrane region. The three detected allelic variants differed only in the stem and transmembrane regions. *NCR2* codes for a functional activating receptor (lysine in the transmembrane region) as well. Three protein variants with minor changes in the signal peptide and the cytoplasmic tail not affecting their potential function were identified. All identified *NCR3* sequences have premature stop codons in the Ig-like domain. The amino acid sequence similarity to camel sequences was 96%.

## Discussion

In contrast to the rather conservative organization of the mammalian major histocompatibility complexes, natural killer cell receptor genes and their complex genomic regions are evolutionarily flexible. Several different types of genomic organization of the NKR regions have been recognized in mammals (Martin et al., 2002; Hao et al., 2006; Guethlein et al., 2015), and sometimes striking differences have been observed between related taxa (Kelley et al., 2005; Sanderson et al., 2014; Schwartz et al., 2017; Schwartz and Hammond, 2018). Therefore, studies of genes encoding NK cell receptors may contribute to our understanding of the heterogeneity of NK cell functions in particular mammalian species. At the same time, these genes and especially complex genomic regions such as NKC and LRC represent a relevant model for evolutionary biology. Characterization of NKR genes in so far poorly studied species and/or families can bring new information on evolutionary mechanisms governing this part of the mammalian immunogenome. Despite the importance of camels as a model for immunogenetic studies (Ciccarese et al., 2014), virtually nothing is known about NK cells in camels in terms of their morphology, functions, their surface receptors, and/or underlying genes. In this context, this study represents the first report on the NKC and LRC genomic regions and on *NCR* genes in Old World camels and their comparison with a New World camelid, the alpaca.

Whole genome sequences of Old World camels, *C. dromedarius*, *C. bactrianus*, and *Camelus ferus* (Jirimutu et al., 2012; Wu et al., 2014; Fitak et al., 2016) and of the alpaca *V. pacos* (NCBI Genome^13^ accession GCA_000164845.3) are currently available. However, their annotation is rather fragmentary and largely composed of predicted sequences generated *in silico*, based on homologies and sequence similarities with other mammalian species. Taking into consideration the quality of resources including the availability of biological material, we focused on the two domestic camel species, *C. dromedarius* and *C. bactrianus*. Even for these species, however, the current status of whole genome assemblies proved to be insufficient for a correct annotation of NKR genes, especially of the LRC, containing multiple copies of sometimes highly similar sequences and exhibiting copy number variations. Therefore, the major resource for our analyses was a new genome assembly of the *C. dromedarius* genome obtained by a combination of several long-read sequencing techniques and bioinformatic approaches (Elbers et al., 2019).

In all genes selected for sequence analyses, the genomic sequences of NKR genes were highly similar in both camel species studied as well as to available alpaca sequences. Such a high sequence similarity was observed for a number of other genes and was also characteristic for MHC class II sequences (Plasil et al., 2016). Therefore, it seems that PCR failures observed in some cases probably do not indicate polymorphisms in the primer binding site. Taking also into consideration the generally low polymorphism of the camel genomes (Fitak et al., 2016), the occurrence of a putative polymorphic variant on both chromosomes is not too likely. Moreover, the PCR failures concerned mostly the loci *KLRC2* and *KIR3DL* in the Bactrians, which seem to be both pseudogenes, so we have not explored them further for the purposes of this study. Nevertheless, both loci merit to be further investigated in the future. Information that the monomorphic status of the Bactrian *KLRC2* could be explained by allelic drop-out or existence of copy number variation, i.e., partial/total deletion of *KLRC2* from some Bactrian NKC haplotypes, and that polymorphic amino acid positions within the *KIR3DL* sequences were concentrated in the second immunoglobulin-like domain, known to interact with MHC class I ligands in mammals and in the stalk region of the molecule, need to be explored.

Another potential technical problem is the use of long-range PCRs for amplifying related members of a gene family, which may produce chimeric products. The NKC genes analyzed here were in majority single (not duplicated) genes with characteristic sequences. The LRC genes analyzed, with only *LILR* genes as members of expanded gene family/families, were different to such an extent that we could distinguish them. In addition, both types of phylogenetic trees clearly supported the individuality of each gene. Moreover, sequences successfully amplified as a whole matched the reference sequences. The remaining ones were amplified in two pieces, and again, they matched the reference sequences. Although we have checked the overlapping sequences of two-piece PCRs and they did not indicate more polymorphisms, we cannot strictly exclude the possibility that such a sequence could be composed of pieces of two different yet highly homologous loci, taking also into consideration numerous fragments of *LILR* genes observed in CamDro2.

The genomic organization of the NKC region of camelids differs from cattle, the phylogenetically most closely related species, whose NKR genes have been studied so far. While in cattle an expansion of *KLRC* and *KLRH* genes was reported (Schwartz et al., 2017), the minimal set of *KLR* genes observed in camelids resembles the genomic organization of the NKC of the domestic pig. Similarly, the leukocyte receptor complex of camels is strikingly different from the cattle LRC containing expanded *KIR* (Sanderson et al., 2014) and *LILR* genes (Hogan et al., 2012). In camels, the LRC with non-expanded *KIR* genes and several pseudogenes seems to be even less complex than in the pig (Schwartz and Hammond, 2018).

Within the natural killer complex, all types of *KLR* genes identified in mammals (Hao et al., 2006) were found. None of them apparently expanded into a large family; the maximum number of members within a family was two. Similar to other mammalian species, the *KLRA* gene codes for a homodimeric type II inhibitory receptor (Ly49) (Dimasi and Biassoni, 2005), *KLRC1* encodes an inhibitory receptor with two ITIMs motifs (NKG2A) (Vance et al., 1998), *KLRC2* encodes an activating receptor (NKG2C) (Lanier et al., 1998), and *KLRG1* codes an inhibitory receptor for cadherin molecules (Ito et al., 2006). These data suggest that the function of these molecules could be very similar to human and other mammalian NK receptors, especially in terms of their capacity to form heterodimers CD94/NKG2A (KLRD/KLRC1), CD94/NKG2C (KLRD/KLRC2) (Braud et al., 1998), and/or KLRE/KLRI (Saether et al., 2008). In humans, the heterodimers CD94/NKG2A (KLRD/KLRC1) and CD94/NKG2C (KLRD/KLRC2) recognize a relatively low polymorphic non-classical MHC class I ligand HLA-E, and their polymorphism is also rather low (Braud et al., 1998). Contrary to rats, in which KLRH recognize MHC class I ligand (Daws et al., 2012), camelid *KLRH* sequences represent only remnants of a full gene sequence. Although mRNA for bovine KLRJ was described (Storset et al., 2003), the precise splicing of camelid *KLRJ* and possible expression as a functional receptor remains to be verified. The low polymorphism of camelid *KLRK* is comparable to the limited polymorphism of this gene in humans and mice encoding an activating receptor NKG2D for diverse ligands (reviewed in Lanier, 2015).

The genomic organization of the NKC is similar in both domestic camel species and in *V. pacos*. The functionally important polymorphism of NKC genes is limited, with one monomorphic gene and six genes with two to three allelic protein variants in *C. dromedarius*. An even higher number of monomorphic *KLR* genes (three functional and two potential pseudogenes) was observed in *C. bactrianus*, and its *KLRC2* seems to be a pseudogene. It is not clear how this low NKC variation can be related to the fact that no “HLA-E-like” molecule has been found to date outside of simians and rodents and to our recent finding that the MHC gene cluster containing *HLA-E* in humans has been lost in camels, similarly to cattle and pigs (Plasil et al., 2019). Interestingly, *V. pacos* seems to be more polymorphic in NKC, at both the genomic and protein levels, despite the limited number of individual genomes analyzed.

Within the LRC region, no *KIR* genes have expanded, while *LILR* genes expanded both activating and inhibitory family members. As for the NKC, the same overall organization of the LRC with *FCAR*, *NCR1*, *KIR*, and *LILRB1* genes, three *LILRB* genes encoding a 4-Ig-like domain receptor, low variable *KIR3DL* and *LILRB1*, and unresolved *LILR* gene fragments was observed in *C. bactrianus*. The polymorphism of *KIR3DL* was similar in the dromedary (seven possible protein variants) and the alpaca (seven functional protein variants), while the alpaca seems to be more variable in the *LILRB1* gene (seven vs. three protein variants, respectively). Unfortunately, we were unable to analyze further alpaca *LILR* genes, mainly due to a lack of correct full-length gene reference sequences and/or to a low coverage of NGS reads available in public databases.

NCR1, NCR2, and NCR3 are the major activating receptors on human NK cells (reviewed in Koch et al., 2013). The *NCR1* gene is located within the LRC; *NCR2* and *3* are located on the human chromosome 6, with *NCR3* mapping within the MHC (Lanier, 2005). The chromosome location of *NCR1*, *NCR2*, and *NCR3* genes in the dromedary corresponds to the human homologues, suggesting an orthologous nature of the *NCR* sequences retrieved. However, only *NCR1* and *NCR2* have a structure of functional genes, while *NCR3* appears to be a pseudogene. The *NCR1*, *NCR2*, and *NCR3* genes of *C. bactrianus* and *V. pacos* are very similar in terms of their genomic locations, sequence homologies, and genomic variation.

We are aware of the limitations due to the quality of the current assembly; however, the clusters of the *LILR* sequences identified in the phylogenetic trees indicated, similarly to NKC genes, the individuality of each of the genes. Although the purpose of this study was to outline the general organization of the two NKR complexes in terms of major gene families represented, and their location within NKC and LRC, further work is needed to definitively resolve the complex structure of LRC region, and a detailed characterization of individual *LILR* genes and pseudogenes is needed.

Taken together, this first report on NKR genes in camelids revealed features characteristic for NKC and LRC of *Tylopoda*. Despite close phylogenetic relationships to cattle, important differences in the NKC and LRC genomic organization and their polymorphism were observed. On the other hand, many similarities with pigs were found. The data presented here increase our genetic knowledge of the innate immune variation in dromedaries and Bactrian camels and contribute to studies of NKR evolution in mammals. The results of this project add to our understanding of specificities of the camel immune system and its functions and represent a prerequisite for future investigations on MHC/NKR interactions in health and disease.

## Data Availability Statement

The camel datasets generated for this study can be found in the NCBI´s GenBank^®^. The sequences obtained for NKC genes have accession numbers MK644262 - MK644413. The LRC gene sequences for both camel species have accession numbers MK644414 - MK644532. The NCRs sequences have accesion numbers MK473784 - MK473840.

## Ethics Statement

The dromedary DNA samples in this study were transferred from previous projects [Austrian Science Fund (FWF) P1084-B17 and P24706-B25; PI: PB] and originated either from plucked hair, EDTA blood collected commensally on Whatman FTA cards (Sigma-Aldrich, Vienna, Austria) during routine veterinary controls or from DNA extracts sent by collaborators under bilateral agreements. Samples were imported with permits from the Austrian Ministry of Labour, Social Affairs, Health and Consumer Protection. All Bactrian camel samples were collected commensally during veterinary procedures for previous research projects (GACR 523/09/1972; PI: PH). All samples were collected by a licensed veterinarian in compliance with all ethical and professional standards.

## Author Contributions

JF made NKC annotation, designed primers, carried out PCR for NKR genes, and analyzed data. JO made all NGS mappings. AJ made LRC annotation and carried out PCR for *LILRB1*. JE provided CamDro2 whole genome sequence and annotation. JW made microsatellite definition and analysis. AK designed the microsatellite project. PB designed the project. PH designed the project and the NKR study. JF and PH drafted the manuscript. JO, AJ, and JW wrote paragraphs. JE and PB edited the manuscript. All authors read, commented on, and approved the final version of the manuscript.

## Funding

This work was supported by the Austrian Science Fund FWF project P29623-B25, by CEITEC-Central European Institute of Technology with research infrastructure supported by the project CZ.1.05/1.1.00/02.0068 financed from European Regional Development Fund and by the Czech National Sustainability Programme NPU LQ1601. The microsatellite study was supported by Internal Grant Agency of the Faculty of Agronomy, Mendel University, in Brno (IGA FA MENDELU No. IP 057/2017).

## Conflict of Interest Statement

The authors declare that the research was conducted in the absence of any commercial or financial relationships that could be construed as a potential conflict of interest.

## Footnotes

1
https://blast.ncbi.nlm.nih.gov

2
https://www.ncbi.nlm.nih.gov/Structure/cdd

3
http://www.cbs.dtu.dk/services/TMHMM

4
https://www.ncbi.nlm.nih.gov/sutils/splign

5
https://www.ncbi.nlm.nih.gov/tools/primer-blast

6
http://www.bioinformatics.babraham.ac.uk/projects/fastqc/

7
http://broadinstitute.github.io/picard/

8
https://sourceforge.net/projects/bbmap/

9
http://repeatmasker.org/cgi-bin/WEBRepeatMasker

10
https://www.ebi.ac.uk/ena

11
https://software.broadinstitute.org/software/igv/

12
https://www.ncbi.nlm.nih.gov/genbank/

13
https://www.ncbi.nlm.nih.gov/genome
