# Supplementary material for: Natural Killer Cell Receptor Genes in Camels: Another Mammalian Model
Source: Front Genet. 2019 Jul 2;10:620. doi: 10.3389/fgene.2019.00620 (PMC6614441; doi:10.3389/fgene.2019.00620)
Supplement: Supplementary file 1 [file DataSheet_1.pdf]

Supplementary Material

Natural killer cell receptor genes in camels: another mammalian model

Jan Futas, Jan Oppelt, April Jelinek, Jean P. Elbers, Jan Wijacki, Ales Knoll, Pamela A. Burger, Petr Horin*

*** Correspondence:** Petr Hořín: horin@dior.ics.muni.cz

Supplementary Table 1. Primers and annealing temperatures used for amplification

Locus	size of amplicon in *C. dromedarius*	forward primer	reverse primer	annealing temperature	PCR protocol
KLRA	12674	5´-GACAGGTAACCGCTCTGCAA-3´	5´-TACGGCTCCATTCCCTCTCA-3´	63°C	A
KLRC1	4902	5´-ACAGTGTTCTTCCACAAATGACC-3´	5´-ATGAAAGATACAGTTACCCAGCA-3´	63°C	C
KLRC2	6677	5´-GAGAAGTACGTTTGGTCATTGATGT-3´	5´-GGTTACCATTAACCACTTTCTGCC-3´	62°C	A
KLRD	4062	5´-GTGGGTGAGGACTTCCTGTG-3´	5´-ATTGCCAACCCCATCTGTCC-3´	63°C	C
KLRE	9886	5´-TCACCGGTATGTGGGTGCTA-3´	5´-CGTCTTCTAAAGTGCATCGGC-3´	58°C	A
KLRI	10345	5´-TACCAGGACTCCTAGCTGCAT-3´	5´-CCCAAGAAATTTGTGGGTGGT-3´	58°C	A
KLRJ	8342	5´-TAACTAGGCCTCTGCTCCCT-3´	5´-CTAGGGGTCCTCTCCCGATT-3´	60°C	A
KLRK	10407	5´-TCCACTCAAGCTTACAAAAGCC-3´	5´-TCGAGGAGAACACTCAGAGA-3´	60°C	A
KIR3DP	4460	5´-GGCACACGGAAGCAGAAATATGA-3´	5´-TCTCGGTACAAGCAACCGAC-3´	65°C	C
	4214	5´-CTACAGCTGCTACGGCTCTC-3´	5´-ACACTACGCTGGTGCTTTGA-3´	65°C	C
KIR3DL	6445	5´-CCCAGGGAGAAGCAGAgAGC-3´	5´-AGAGGCTGATGACGAGAGGT-3´	63°C	D
	6792	5´-CTCCTTCACATCCTGCCTGG-3´	5´-TGAAACACCCTTCGCAGAGC-3´	63°C	D
LILRA 2-Ig	7220	5´-GTTACTGTCTTCACAAGTCTCCG-3´	5´-CTGTAAATGCATCAACATCTCTCA-3´	58°C	B
LILRA 4-Ig	4327	5´-TGGACTCCAGTCATCCTCCTAT-3´	5´-TGAATGGATTGATAGGGCCAGG-3´	58°C	B
LILRB1	5299	5´-ACTGTCTGGCACCGTATAGC-3´	5´-GGCCGACCATGTGTAGGAAT-3´	63°C	B
	5734	5´-TCCTTCTGCTTCGACCCAAC-3´	5´-CCTCCAAACGTACACCCTCC-3´	63°C	B
LILRB2	8010	5´-AGACCTCTAACCCAAATGAATGC-3´	5´-CTTTGATTGGGAATTTGGGATTGG-3´	58°C	B
LILRB2-like	7040	5´-GTGTGCTGAGGGCACTGTAT-3´	5´-CAGCAGGAGTGCACATTTCG-3´	58°C	B
LILRB3	4097	5´-CCTCCTGTCCCTTTGCTCAG-3´	5´-AGTACAGTGTCAGCGTCACC-3´	58°C	B
	5142	5´-GACCCTGCTGTGTCAGTCAA-3´	5´-TGGAAGCAACCACGGATGAA-3´	58°C	B
NCR1	3578	5´-ACAGGTGTGAGCTTCAGGTG-3´	5´-ACACGTGCCACAAACACAAG-3´	68°C	C
	3758	5´-ATCCTTGTGTTTGTGGCACG-3´	5´-GGAGTCTCACGTTCCAAGCA-3´	68°C	C
NCR2	7586	5´-ACACACGGTGACACACCTTT-3´	5´-ACAGAGGTGTTGCCAAGAGG-3´	65°C	B
	7462	5´-TCCAGGCCCACTATGTCTCA-3´	5´-GCAGGTGATCTGCTGACAGT-3´	65°C	B
NCR3	3040	5´-TGGATGGCGTCAGCTAATCC-3´	5´-GCTCCTCTGAAGGCCAGAAA-3´	65°C	E

Supplementary Table 2. PCR protocols

**PCR protocol A**		
5x Expand LR buffer	2.5 µl	thermocycler program:
10mM dNTPs (each)	0.625 µl	92°C 2 min
10µM forward primer	0.5 µl	10 rounds of [92°C 10 sec; annealing 20 sec; 68°C 1 min per kb]
10µM reverse primer	0.5 µl	25 rounds of [92°C 10 sec; annealing 15 sec; 68°C 1 min per kb+20 sec per cycle]
Expand Long Range Enzyme 5U/µl	0.175 µl	68°C 7 min
H_2O	add to 12.5 µl	hold at 8°C
genomic DNA	100 ng	
**PCR protocol B**		
5x Expand LR buffer	2.5 µl	thermocycler program:
10mM dNTPs (each)	0.625 µl	92°C 2 min
10µM forward primer	0.5 µl	10 rounds of [92°C 10 sec; annealing 20 sec; 68°C 1 min per kb]
10µM reverse primer	0.5 µl	25 rounds of [92°C 10 sec; annealing 15 sec; 68°C 1 min per kb+20 sec per cycle]
Expand Long Range Enzyme 5U/µl	0.175 µl	68°C 7 min
DMSO	0.375 µl	hold at 8°C
H_2O	add to 12.5 µl	
genomic DNA	100 ng	
**PCR protocol C**		
5x KAPA A buffer	2.5 µl	thermocycler program:
5x KAPA Enhancer	2.5 µl	95°C 3 min
10mM dNTPs (each)	0.25 µl	35 rounds of [95°C 25 sec; annealing 15 sec; 72°C 30 sec per kb]
10µM forward primer	0.625 µl	72°C 1 min per kb
10µM reverse primer	0.625 µl	hold at 8°C
KAPA 2GO Polymerase 5U/µl	0.1 µl	
H_2O	add to 12.5 µl	
genomic DNA	50 ng	
**PCR protocol D**		
5x KAPA LR buffer	2.5 µl	thermocycler program:
25mM $MgCl_2$	0.875 µl	94°C 3 min
10mM dNTPs (each)	0.375 µl	35 rounds of [94°C 25 sec; annealing 15 sec; 72°C 1 min per kb]
10µM forward primer	0.625 µl	72°C 1 min per kb
10µM reverse primer	0.625 µl	hold at 8°C
KAPA Long Range Polymerase 2,5U/µl	0.1 µl	
H_2O	add to 12.5 µl	
genomic DNA	100 ng	
**PCR protocol E**		
2x PCRBio HS Taq MixRed	6.25 µl	thermocycler program:
10µM forward primer	0.5 µl	95°C 2 min
10µM reverse primer	0.5 µl	40 rounds of [95°C 15 sec; annealing 15 sec; 72°C 15 sec per kb]
H_2O	add to 12.5 µl	72°C 2 min
genomic DNA	50 ng	hold at 8°C

Supplementary Table 3. List of samples

Lab number	Place of collection	Country of origin	Year of collection	Sample type	Sex	Age (years)
C. dromedarius 415	Jordan, Irbid	Qatar	2010	hair plucked	F	10
C. dromedarius 418	Jordan, Irbid	Qatar	2010	hair plucked	F	6
C. dromedarius 668	Iran, Ahwaz	Iran	2011	DNA extract	UK	UK
C. dromedarius 795A	Saudi Arabia, Al Jouf	Saudi Arabia	2013	FTA card	F	UK
C. dromedarius 799A	Saudi Arabia, Al Jouf	Saudi Arabia	2013	FTA card	M	UK
C. dromedarius 800A	Saudi Arabia, Al Jouf	Saudi Arabia	2013	FTA card	M	UK
C. dromedarius 801A	Austria, Eithental	Canary Islands	2013	EDTA blood	F	UK
C. dromedarius 804A	UAE, Dubai	UAE	2013	FTA card	F	UK
C. dromedarius 805A	Kenya, Pokot	Kenya	2013	FTA card	F	UK
C. dromedarius 815A	Sudan, Karthoum	Sudan	2013	FTA card	F	8
C. dromedarius 818A	Pakistan	Pakistan	2013	FTA card	M	UK
C. dromedarius 852	Nigeria	Nigeria	2013	FTA card	UK	UK
C. dromedarius 890	Kazakstahn, west	Kazakhstan	2015	FTA card	UK	UK
C. dromedarius 891	Kazakstahn, west	Kazakstahn	2015	FTA card	UK	UK
C. dromedarius 893	Kazakstahn, west	Kazakstahn	2015	FTA card	UK	UK
C. bactrianus 158	Austria	Austria	2013	EDTA blood	F	UK
Bactrian_35	Mongolia, Norovlin	Mongolia	2009	FTA card	M	8
Bactrian_53	Mongolia, Norovlin	Mongolia	2009	FTA card	M	7
Bactrian_56	Mongolia, Norovlin	Mongolia	2009	FTA card	M	8
Bactrian_159	Mongolia, Bayan Ovoo	Mongolia	2009	FTA card	F	7
Bactrian_176	Mongolia, Bayan Ovoo	Mongolia	2009	FTA card	M	5
Bactrian_186	Mongolia, Bayan Ovoo	Mongolia	2009	FTA card	M	10
Bactrian_191	Mongolia, Bayan Ovoo	Mongolia	2009	FTA card	M	3
Bactrian_222	Mongolia, Galshar	Mongolia	2009	FTA card	F	4
Bactrian_253	Mongolia, Galshar	Mongolia	2009	FTA card	F	8
Bactrian_259	Mongolia, Galshar	Mongolia	2009	FTA card	M	6

Supplementary Table 4. Estimation of genetic variability in panels of species

Locus	species	number of individuals	size of genomic sequence	No. of SNPs in genomic sequence	frequency of SNPs in genomic sequence	size of coding sequence	No. of SNPs in coding sequence	frequency of SNPs in coding sequence	number of predicted mRNA haplotypes	number of predicted proteins
KLRA	*C. dromedarius*	8	12674	20	0.16%	828	2	0.24%	3	2
	C. bactrianus	10	12678	100	0.79%	828	11	1.33%	3	2
	Vicugna pacos	4	12555	144	1.15%	828	15	1.81%	5	5
KLRC1	*C. dromedarius*	10	4902	27	0.55%	711	6	0.84%	4	3
	C. bactrianus	10	4902	27	0.55%	711	4	0.56%	6	3
	Vicugna pacos	4	4909	67	1.36%	711	5	0.70%	6	5
KLRC2	*C. dromedarius*	8	6862	27	0.39%	597	2	0.34%	2	2
	C. bactrianus	6	6862	0	0.00%	597	0	0.00%	1	1
	Vicugna pacos	4	6876	84	1.22%	597	9	1.51%	5	5
KLRD	*C. dromedarius*	10	4059	16	0.39%	540	4	0.74%	5	3
	C. bactrianus	10	4059	12	0.30%	540	1	0.19%	4	1
	Vicugna pacos	4	4056	24	0.59%	540	3	0.56%	4	3
KLRE	*C. dromedarius*	10	9902	17	0.17%	771	0	0.00%	2	2
	C. bactrianus	10	9902	16	0.16%	753	1	0.13%	2	1
	Vicugna pacos	4	8118	88	1.08%	753	12	1.59%	5	5
KLRI	*C. dromedarius*	7	10332	16	0.15%	747	1	0.13%	2	1
	C. bactrianus	10	10332	19	0.18%	747	0	0.00%	1	1
	Vicugna pacos	4	9702	67	0.69%	747	3	0.40%	4	3
KLRJ	*C. dromedarius*	9	8339	12	0.14%	855	2	0.23%	3	2
	C. bactrianus	10	8337	18	0.22%	855	0	0.00%	2	1
	Vicugna pacos	4	8067	82	1.02%	855	11	1.29%	5	5
KLRK	*C. dromedarius*	8	10407	18	0.17%	645	2	0.31%	3	2
	C. bactrianus	9	10407	25	0.24%	645	3	0.47%	3	2
	Vicugna pacos	4	9970	85	0.85%	645	3	0.47%	4	4
KIR3DP	*C. dromedarius*	9	8261	15	0.18%					
	C. bactrianus	10	8261	56	0.68%					
	Vicugna pacos	4	8357	92	1.10%					
KIR3DL	*C. dromedarius*	8	13061	46	0.35%	1375	7	0.51%	7	7
	C. bactrianus	4	13061	124	0.95%	1375	24	1.75%	3	3
	Vicugna pacos	4	13469	207	1.54%	1377	18	1.31%	7	7
LILRB1	*C. dromedarius*	9	9843	71	0.72%	1422	8	0.56%	10	3
	C. bactrianus	10	9843	4	0.04%	1422	0	0.00%	4	1
	Vicugna pacos	4	9893	124	1.25%	1425	22	1.54%	8	7
LILRA 2-Ig	*C. dromedarius*	9	7220	32	0.44%	1074	5	0.47%	7	3
	C. bactrianus	9	7210	1	0.01%	1074	0	0.00%	2	1
LILRA 4-Ig	*C. dromedarius*	9	4451	10	0.22%	1479	1	0.07%	4	3
	C. bactrianus	6	4442	33	0.74%	1479	13	0.88%	6	4
LILRB2	*C. dromedarius*	7	8010	20	0.25%	1650	3	0.18%	5	6
	C. bactrianus	8	7994	22	0.28%	1644	1	0.06%	2	2
LILRB2-like	*C. dromedarius*	10	7078	14	0.20%	1650	3	0.18%	6	6
LILRB3	*C. dromedarius*	9	8061	21	0.26%	1896	10	0.53%	5	4
	C. bactrianus	8	8094	55	0.68%	1896	18	0.95%	6	5
NCR1	*C. dromedarius*	10	7212	69	0.96%	969	2	0.21%	6	2
	C. bactrianus	10	7215	26	0.36%	969	6	0.62%	5	3
	Vicugna pacos	4	7248	71	0.98%	969	5	0.52%	7	3
NCR2	*C. dromedarius*	10	14723	58	0.39%	903	2	0.22%	3	2
	C. bactrianus	8	14723	38	0.26%	903	5	0.55%	3	3
	Vicugna pacos	4	13134	85	0.65%	903	5	0.55%	4	3
NCR3	*C. dromedarius*	9	3040	8	0.26%	561	1	0.18%	2	2
	C. bactrianus	10	3040	11	0.36%	561	1	0.18%	2	2
	Vicugna pacos	4	3036	11	0.36%	561	3	0.53%	3	3

presumably pseudogene

Supplementary Table 5. Amino acid sequence similarity of dromedary NK receptors with their orthologues in Bactrian camel, alpaca, cattle and pig

Camelus dromedarius	*Camelus bactrianus*	*Vicugna pacos*	*Bos taurus*	*Sus scrofa*	BLASTP
KLRA	97% - 100%	88% - 89%	58%	58%	identities
	98% - 100%	91%	69%	68%	positives
	§	§	NP_776801.1	NP_999503.1	reference
KLRC1	99% - 100%	97% - 98%	62% - 69%	69%	identities
	99% - 100%	97% - 98%	72% - 80%	82%	positives
	§	§	e.g. NP_001162059.1	XP_020948016.1	reference
KLRC2	98%	94% - 95%	60% - 61%	0%	identities
	98%	96%	73% - 75%	0%	positives
	§	§	e.g. NP_001091632.1	*	reference
KLRD	100%	96%-97%	64% - 69%	73%	identities
	100%	97%	73% - 77%	85%	positives
	§	§	e.g. XP_005207144.1	XP_005655734.2	reference
KLRE	96% - 99%	89% - 93%	74%	72%	identities
	96% - 99%	90% - 94%	82%	81%	positives
	§	§	XP_005207145.1	XP_005655736.2	reference
KLRI	99%	96%	65%	67%	identities
	99%	96% - 97%	79%	80%	positives
	§	§	XP_005207101.1	XP_020948014.1	reference
KLRJ	99%-100%	96%	70% -71%	71% - 72%	identities
	100%	97% - 98%	81%	83%	positives
	§	§	NP_001002884.1	XP_013843835.1	reference
KLRK	99%	93% - 94%	72% - 73%	70%	identities
	99%-100%	94% - 95%	81%	79%	positives
	§	§	NP_001068607.1	XP_005655731.1	reference
KIR3DL	97% - 99%	92% - 93%	45% - 47%	59%	identities
	97% - 99%	93% - 94%	56% -58%	70%	positives
	§	§	e.g. NP_852116.1	NP_001106689.1	reference
LILRB1	99%	93%	59%	68%	identities
	99%	93% - 94%	68%	74%	positives
	§	§	* XP_024834567.1	* XP_013854218.2	reference
LILRB2	92% - 93%	ND	63% - 67%	50% - 69%	identities
	95%	ND	71% - 74%	61% -76%	positives
	§	ND	e.g. XP_024834575.1	e.g. XP_020950636.1	reference
LILRB3	96% - 97%	ND	63% - 64%	65%	identities
	97% - 98%	ND	72% - 73%	72%	positives
	§	ND	e.g. XP_024844763.1	* XP_020950636.1	reference
LILRA 2-Ig	99% - 100%	ND	0%	0%	identities
	100%	ND	0%	0%	positives
	§	ND	*	*	reference
LILRA 4-Ig	94% - 100%	ND	67% - 72%	65% - 73%	identities
	94% - 100%	ND	75% - 80%	72% - 80%	positives
	§	ND	e.g. XP_024844775.1	e.g. NP_001121923.1	reference
NCR1	99%	95%	71%	71%	identities
	99%	96%	81%	79%	positives
	§	§	NP_899209.1	NP_001116615.1	reference
NCR2	99% - 100%	95% - 96%	0%	65%	identities
	99% - 100%	95% - 96%	0%	72%	positives
	§	§	*	XP_020954329.1	reference
NCR3	99% - 100%	96% - 97%	69%	44%	identities
	99% - 100%	97% - 98%	77%	48%	positives
	§	§	NP_001035614.1	XP_013833084.1	reference

§ - this study sequences; * - not present in genome/ mostly related sequence

ND – not determined; presumably pseudogene

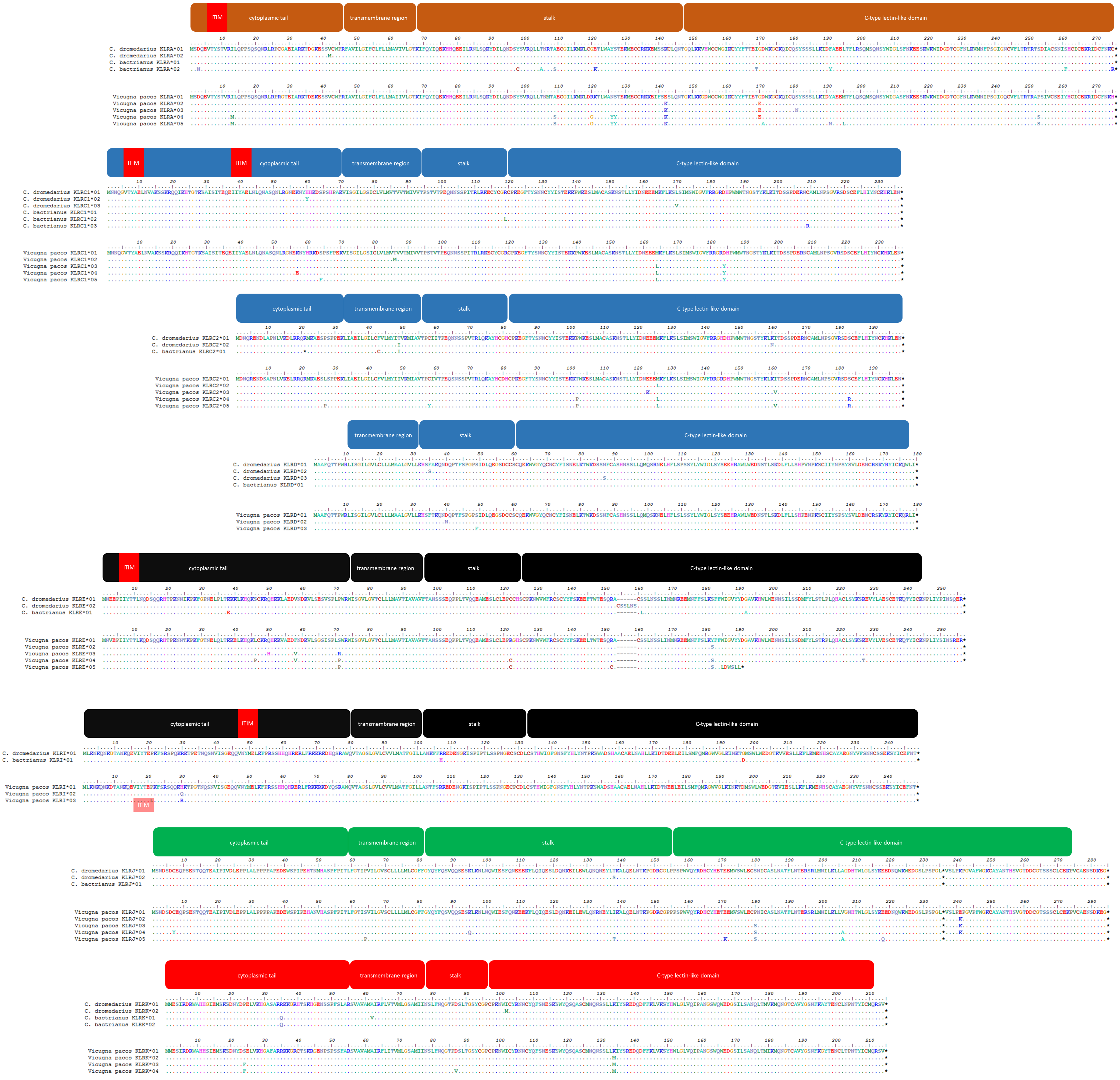

Supplementary Figure S1. Alignments of predicted allelic variants of natural killer complex receptors. Domain organization of protein molecule is depicted as color rectangles. ITIM – signaling motif, *dot* – the same amino acid, *dash* – gap in alignment, *asterisk* – stop codon in coding sequence

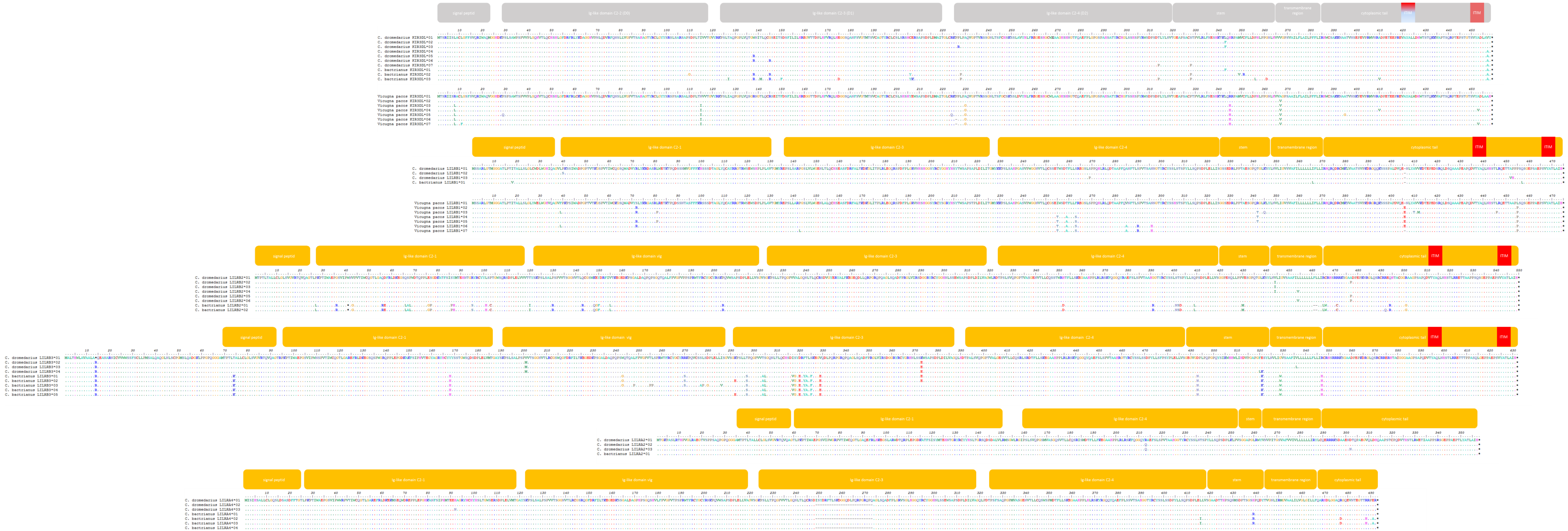

Supplementary Figure S2. Alignments of predicted allelic variants of leukocyte receptor complex receptors. Domain organization of protein molecule is depicted as color rectangles. ITIM – signaling motif, X- codon with 2-bp insertion to restore reading frame of KIR3DL mRNA, *dot* – the same amino acid, *dash* – gap in alignment, *asterisk* – stop codon in coding sequence

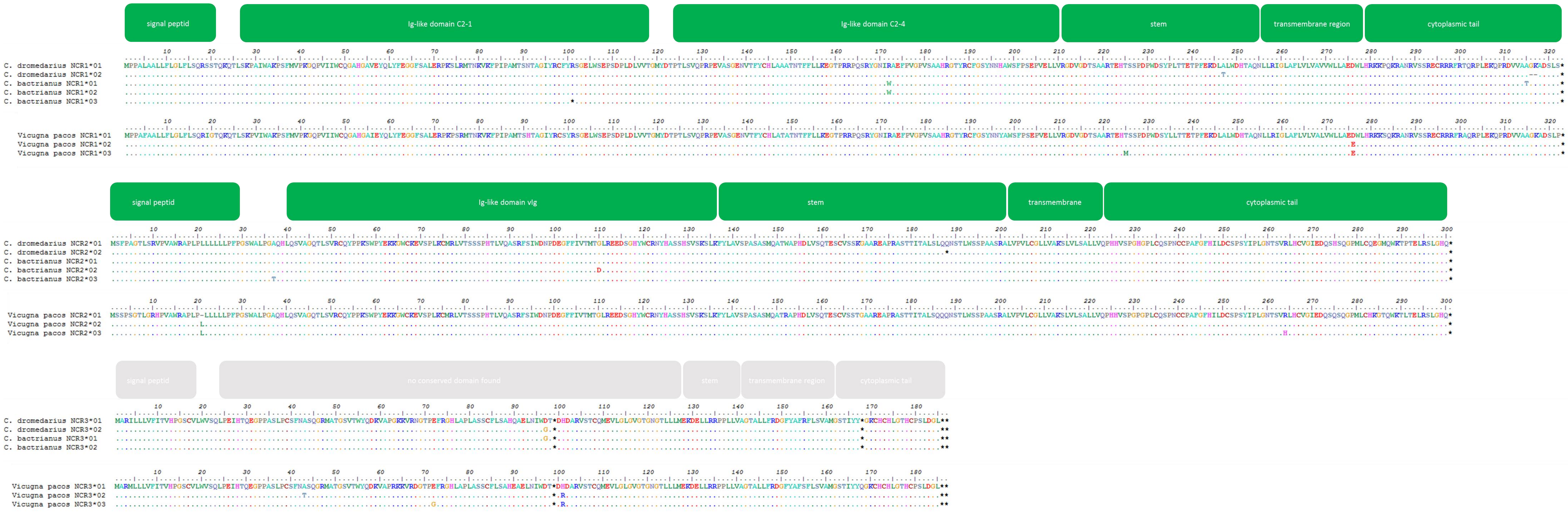

Supplementary Figure S3. Alignments of predicted allelic variants of NCR receptors. Domain organization of protein molecule is depicted as color rectangles. *dot* – the same amino acid, *dash* – gap in alignment, *asterisk* – stop codon in coding sequence
